# Supplementary material for: Modeling glioblastoma heterogeneity as a dynamic network of cell states
Source: Mol Syst Biol. 2021 Sep 16;17(9):e10105. doi: 10.15252/msb.202010105 (PMC8444284; doi:10.15252/msb.202010105)
Supplement: Supplementary file 5 — Source Data for Figure 3 [file MSB-17-e10105-s001.zip › Figure3A_sourcedata/GSEA_3065/hallmarks_state5.GseaPreranked.1623416302988/HALLMARK_KRAS_SIGNALING_UP.html]

Details for gene set HALLMARK\_KRAS\_SIGNALING\_UP[GSEA]

|  || Dataset | state5 |
| Phenotype | NoPhenotypeAvailable |
| Upregulated in class | na\_pos |
| GeneSet | HALLMARK\_KRAS\_SIGNALING\_UP |
| Enrichment Score (ES) | 0.280718 |
| Normalized Enrichment Score (NES) | 1.1185895 |
| Nominal p-value | 0.2 |
| FDR q-value | 0.26755136 |
| FWER p-Value | 0.928 |
Table: GSEA Results Summary

  

Fig 1: Enrichment plot: HALLMARK\_KRAS\_SIGNALING\_UP      
 Profile of the Running ES Score & Positions of GeneSet Members on the Rank Ordered List

  

| PROBE | GENE SYMBOL | GENE\_TITLE | RANK IN GENE LIST | RANK METRIC SCORE | RUNNING ES | CORE ENRICHMENT || 1 | PLAT |  |  | 37 | 0.714 | 0.0555 | Yes |
| 2 | RBM4 |  |  | 149 | 0.482 | 0.0833 | Yes |
| 3 | EMP1 |  |  | 258 | 0.404 | 0.1050 | Yes |
| 4 | GPNMB |  |  | 281 | 0.393 | 0.1353 | Yes |
| 5 | CXCR4 |  |  | 450 | 0.325 | 0.1436 | Yes |
| 6 | CPE |  |  | 624 | 0.280 | 0.1476 | Yes |
| 7 | ADAM17 |  |  | 711 | 0.259 | 0.1595 | Yes |
| 8 | SPP1 |  |  | 724 | 0.256 | 0.1796 | Yes |
| 9 | CBR4 |  |  | 747 | 0.251 | 0.1981 | Yes |
| 10 | GPRC5B |  |  | 838 | 0.234 | 0.2075 | Yes |
| 11 | DCBLD2 |  |  | 863 | 0.230 | 0.2241 | Yes |
| 12 | PTBP2 |  |  | 914 | 0.222 | 0.2371 | Yes |
| 13 | AVL9 |  |  | 1119 | 0.196 | 0.2305 | Yes |
| 14 | AKT2 |  |  | 1129 | 0.195 | 0.2458 | Yes |
| 15 | PCSK1N |  |  | 1162 | 0.190 | 0.2580 | Yes |
| 16 | TFPI |  |  | 1278 | 0.175 | 0.2597 | Yes |
| 17 | KIF5C |  |  | 1422 | 0.160 | 0.2570 | Yes |
| 18 | SEMA3B |  |  | 1604 | 0.141 | 0.2484 | Yes |
| 19 | FUCA1 |  |  | 1611 | 0.141 | 0.2595 | Yes |
| 20 | SPARCL1 |  |  | 1663 | 0.136 | 0.2652 | Yes |
| 21 | CROT |  |  | 1731 | 0.131 | 0.2686 | Yes |
| 22 | ERO1A |  |  | 1804 | 0.127 | 0.2711 | Yes |
| 23 | MMP11 |  |  | 1813 | 0.126 | 0.2807 | Yes |
| 24 | IGFBP3 |  |  | 2037 | 0.109 | 0.2648 | No |
| 25 | TOR1AIP2 |  |  | 2183 | 0.100 | 0.2568 | No |
| 26 | F2RL1 |  |  | 2256 | 0.094 | 0.2566 | No |
| 27 | KCNN4 |  |  | 2380 | 0.087 | 0.2501 | No |
| 28 | AKAP12 |  |  | 2478 | 0.081 | 0.2459 | No |
| 29 | SDCCAG8 |  |  | 2540 | 0.077 | 0.2455 | No |
| 30 | ZNF277 |  |  | 2564 | 0.076 | 0.2492 | No |
| 31 | NIN |  |  | 2601 | 0.074 | 0.2513 | No |
| 32 | GADD45G |  |  | 2682 | 0.069 | 0.2481 | No |
| 33 | PEG3 |  |  | 2686 | 0.069 | 0.2536 | No |
| 34 | LY96 |  |  | 2796 | 0.063 | 0.2466 | No |
| 35 | PLAUR |  |  | 2826 | 0.061 | 0.2484 | No |
| 36 | ETV1 |  |  | 2867 | 0.059 | 0.2489 | No |
| 37 | AMMECR1 |  |  | 2897 | 0.058 | 0.2505 | No |
| 38 | ETV5 |  |  | 2957 | 0.055 | 0.2484 | No |
| 39 | EPHB2 |  |  | 3015 | 0.051 | 0.2463 | No |
| 40 | HDAC9 |  |  | 3051 | 0.049 | 0.2465 | No |
| 41 | ANKH |  |  | 3098 | 0.047 | 0.2452 | No |
| 42 | USP12 |  |  | 3215 | 0.040 | 0.2356 | No |
| 43 | PLAU |  |  | 3407 | 0.031 | 0.2167 | No |
| 44 | FBXO4 |  |  | 3586 | 0.024 | 0.1987 | No |
| 45 | DNMBP |  |  | 3660 | 0.022 | 0.1923 | No |
| 46 | WDR33 |  |  | 3664 | 0.021 | 0.1938 | No |
| 47 | TNFRSF1B |  |  | 3727 | 0.019 | 0.1884 | No |
| 48 | NRP1 |  |  | 3735 | 0.019 | 0.1892 | No |
| 49 | TSPAN13 |  |  | 3913 | 0.011 | 0.1702 | No |
| 50 | ATG10 |  |  | 3998 | 0.007 | 0.1614 | No |
| 51 | MAP3K1 |  |  | 4259 | -0.004 | 0.1325 | No |
| 52 | MTMR10 |  |  | 4266 | -0.004 | 0.1322 | No |
| 53 | VWA5A |  |  | 4356 | -0.008 | 0.1229 | No |
| 54 | SATB1 |  |  | 4505 | -0.014 | 0.1074 | No |
| 55 | CCND2 |  |  | 4539 | -0.016 | 0.1050 | No |
| 56 | BMP2 |  |  | 4770 | -0.025 | 0.0812 | No |
| 57 | CBL |  |  | 4814 | -0.026 | 0.0786 | No |
| 58 | BTBD3 |  |  | 4891 | -0.029 | 0.0725 | No |
| 59 | CA2 |  |  | 5139 | -0.038 | 0.0479 | No |
| 60 | G0S2 |  |  | 5284 | -0.043 | 0.0353 | No |
| 61 | PPP1R15A |  |  | 5321 | -0.045 | 0.0351 | No |
| 62 | SCN1B |  |  | 5371 | -0.048 | 0.0335 | No |
| 63 | STRN |  |  | 5405 | -0.049 | 0.0339 | No |
| 64 | PRDM1 |  |  | 5742 | -0.063 | 0.0014 | No |
| 65 | ZNF639 |  |  | 6107 | -0.080 | -0.0328 | No |
| 66 | CCSER2 |  |  | 6527 | -0.103 | -0.0712 | No |
| 67 | RGS16 |  |  | 6543 | -0.104 | -0.0642 | No |
| 68 | ETV4 |  |  | 6761 | -0.118 | -0.0787 | No |
| 69 | YRDC |  |  | 6818 | -0.122 | -0.0748 | No |
| 70 | GLRX |  |  | 6822 | -0.123 | -0.0649 | No |
| 71 | PRRX1 |  |  | 7057 | -0.142 | -0.0793 | No |
| 72 | EVI5 |  |  | 7077 | -0.143 | -0.0694 | No |
| 73 | PSMB8 |  |  | 7143 | -0.149 | -0.0642 | No |
| 74 | DUSP6 |  |  | 7258 | -0.162 | -0.0635 | No |
| 75 | ID2 |  |  | 7333 | -0.171 | -0.0575 | No |
| 76 | BPGM |  |  | 7438 | -0.185 | -0.0537 | No |
| 77 | SPRY2 |  |  | 7461 | -0.187 | -0.0405 | No |
| 78 | PRELID3B |  |  | 7556 | -0.198 | -0.0345 | No |
| 79 | TRIB2 |  |  | 7741 | -0.224 | -0.0365 | No |
| 80 | GYPC |  |  | 8050 | -0.285 | -0.0473 | No |
| 81 | PCP4 |  |  | 8304 | -0.356 | -0.0460 | No |
| 82 | SOX9 |  |  | 8327 | -0.361 | -0.0182 | No |
| 83 | TMEM158 |  |  | 8499 | -0.434 | -0.0012 | No |
| 84 | GNG11 |  |  | 8815 | -0.665 | 0.0190 | No |
Table: GSEA details [plain text format]

  

Fig 2: HALLMARK\_KRAS\_SIGNALING\_UP: Random ES distribution      
 Gene set null distribution of ES for **HALLMARK\_KRAS\_SIGNALING\_UP**
